# Wireless Battery-Free Harmonic Communication System for Pressure Sensing

**DOI:** 10.3390/mi11121043

**Published:** 2020-11-27

**Authors:** Deepak Kumar, Saikat Mondal, Yiming Deng, Premjeet Chahal

**Affiliations:** Department of Electrical and Computer Engineering, Michigan State University, East Lansing, MI 48824, USA; mondalsa@msu.edu (S.M.); dengyimi@egr.msu.edu (Y.D.); chahal@egr.msu.edu (P.C.)

**Keywords:** passive, pipeline, pressure, reactive impedance sensor, structural health monitoring, wireless

## Abstract

In this paper, an efficient passive wireless harmonic communication system is proposed for the real-time monitoring of the pressurized pipelines. A pressure sensor is fabricated using the additive manufacturing technique and a harmonic radio frequency (RF) tag is designed to operate at the fundamental frequency (*f${}_{o}$*) of 2 GHz that shifts the phase of the back reflected RF signal according to the applied pressure ranging from 0 to 20 psi. A power efficient phase modulation with virtually no losses is achieved using a hybrid coupler-based phase shifter that efficiently reflect back the incoming signal using an end coupled reactive impedance element/sensor. The phase delay introduced by the reactive element gets doubled with the second harmonic communication, which increases the sensitivity by a factor of two. The concept of harmonic backscattering is exploited to reduce the effects of multi-path interference and self jamming, as well as improving the signal-to-noise ratio (SNR).

## 1. Introduction

Pipelines are the safest and economically viable arterial networks for transporting natural gases and oils across the globe. The pipeline network expands at a faster rate due to the projected growth in population and increased rate of urbanization, creating a demand in supply of natural gas for domestic and industrial use [1]. The natural gases are gathered, transported, and distributed at various pressure levels via metals, composites, and plastic pipelines. Any material subjected to a high or a low cyclic pressure over a prolonged period of time induces a stress on the material. This stress over time compromises the safety and reliability of the pipeline and may lead to a catastrophic failure [2,3,4]. In order to prevent such failures, there is a growing need for the development of economical, real-time, scalable, structural integrity monitoring, and sensing system for the pipeline infrastructure implemented in refineries, chemical plants and manufacturing facilities.

### Related Work

In literature, a number of structural health monitoring (SHM) techniques exist to evaluate the integrity of pipes aiding in improving safety and lifespan of the pipeline infrastructure [5,6]. The different indicators that are monitored for elucidating the pipeline health are cyclic pressure, surface temperature, humidity, and vibration [5,6]. Among these indicators, pressure is the most important and found to be the primary cause of damage to the pipelines [7].

A number of techniques have been proposed to monitor the pipeline pressure; such as piezo based resistive sensors, micro-electromechanical systems (MEMS)-based capacitive sensors, and fiber Bragg grating (FBG)-based negative pressure sensors [7,8,9,10]. The fabrication process of all the sensors is very complex, which either requires a hefty clean room process or very expensive tools for precision. For example, the MEMS-based capacitive sensors require expensive substrate material, chemical etching, wire bonding, and separate housing [10]. Additive manufacturing is a viable alternative of this process, which has been previously proven for electronic system and component design [11]. Three-dimensional printing technique was previously used for designing multiple components in a pressure sensor [12,13,14]. However, a fully 3D-printed pressure sensor is still a feat to achieve. Most of the published research is focused on various other applications and gas pipeline is not the primary focus, which leads to over complexity, limited communication range, and scalability issues.

One of the major limitations of these sensor units is that a direct wired connection is required for data acquisition. The direct probing or wired connection limits the application of the sensors and poses a problem for inaccessible environments, like under ground buried pipelines or large infrastructure with a requirement of thousands of sensing nodes.

A number of wireless sensing approaches have been proposed as an alternate solution to overcome the disadvantages of the wired sensing methods [15,16]. The wireless methods monitor the pressure in the pipeline by deploying a large number of active sensor nodes. The integrated nodes on the surface of the pipes transmit the data wirelessly to the interrogator. The disadvantage of active sensors is the need for an on-board battery, charging and/or replacement requirements increase the implementation and maintenance cost of the system [15,16,17]. Battery-free, low-cost, real-time sensing is ideal to provide a robust and economical system with longer life span and lower maintenance [18,19].

Inductor-capacitor (LC) resonator-based passive sensor tags have been proposed in literature for monitoring the pressure based on capacitive loading, which in turn shifts the resonance frequency [20]. LC resonant sensors operate in near-field configuration and have a limited interrogation distance [21]. The conventional far-field passive pressure sensors operate at a single frequency, which are prone to clutter due to multi-path interference and have a lower signal-to-noise (SNR) due to self-jamming [18]. To overcome these limitations, the harmonic communication approach is desired, that eliminates self jamming, reduces multi-path interference, lowers down phase noise, and increases the SNR. Harmonic-based sensors have been reported in literature for sensing temperature, and pH [22,23]. In this work, a passive harmonic RF tag with integrated pressure sensor is presented for the first time for the real-time monitoring of the pipeline infrastructure.

**Figure 1:**
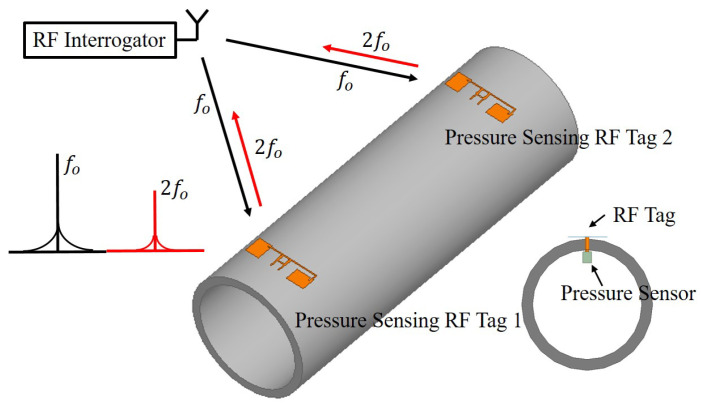
Proposed passive wireless sensing system for the pipeline infrastructure.

In this paper, a harmonic RF system is proposed for gas pipeline infrastructure that consists of an RF tag coupled with a pressure sensor and an RF interrogator. The harmonic RF sensor tag has a receiver (2 GHz) and transmitter (4 GHz) antenna, a harmonic doubler, and an integrated pressure sensor with the phase shifter. The integrated pressure sensor is designed with a cavity based parallel plate reactive impedance element/sensor that changes its reactance with the change in applied pressure from 0–20 psi. The designed sensor is fabricated using the additive manufacturing technique, which uses thin liquid crystalline polymer (LCP) substrate as its diaphragm. The phase shifter translates the change in reactance to the change in received 2 GHz signal’s phase, with the power efficient modulation technique, which is backscattered at harmonic frequency of 4 GHz. A schematic of the proposed sensing system over the pipeline infrastructure is shown in Figure 1.

## 2. Design and Setup

The design of passive wireless harmonic system includes: (A) 3D printed pressure sensor (B) passive harmonic RF sensor tag, and (C) harmonic RF interrogator.

### 2.1. 3D Printed Pressure Sensor

**Figure 2:**
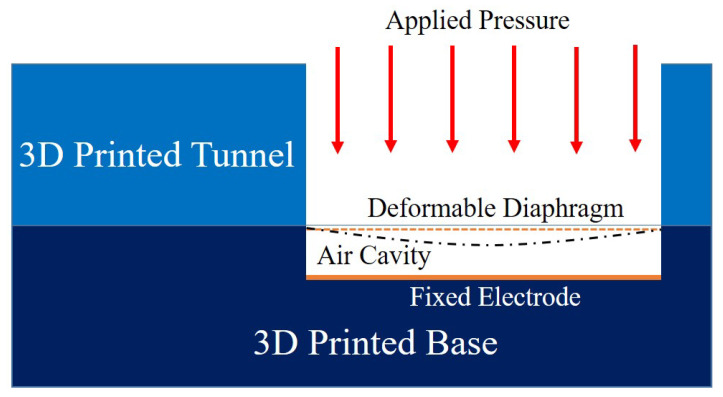
Air cavity-based fully 3D printed pressure sensor.

The designed pressure sensor is an air cavity based parallel plate capacitor, where one electrode has a fixed position and second electrode is deformable due to the pressure as shown in Figure 2. The air cavity in between electrode acts as dielectric medium and holds 1 atmospheric pressure or reference pressure. The pressure is applied through a tunnel directly onto the diaphragm with copper electrode.

**Figure 3:**
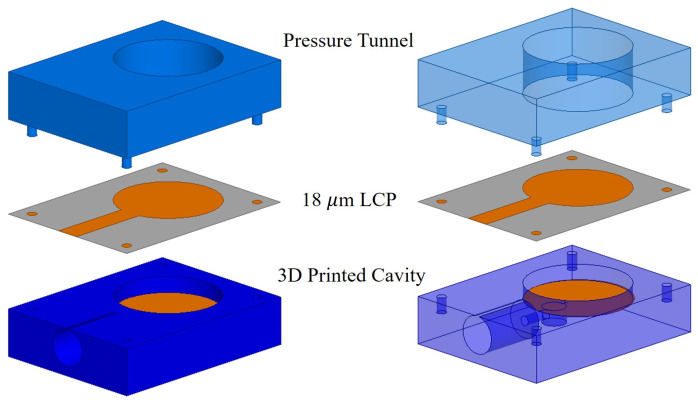
Exploded view of 3D printed pressure sensor with three main components.

The pressure sensor is fabricated in three parts, as shown in Figure 3. The first component has the air cavity with the fixed electrode and electrical access holes for rigid coaxial cable. The copper/ fixed electrode is plated into the cavity’s bottom of the 3D printed part. The second component is the deformable electrode, which is realized using 18 μm-thick LCP film with copper plating. The LCP film is fixed on top of the cavity, such that it makes an electrical connection with the outer shell of the rigid coaxial cable. The third component is a pressure tunnel that directs the pressure onto the electrodes and hold the LCP film in its place. The top part has four alignment legs, which perfectly fits with the bottom component. An extra layer of polymer resin is coated on top of the fully assembled pressure sensor and cured under ultraviolet (UV) light, for eliminating any pressure leaks into or from the air cavity and acts as a seal of the pressure sensor. The outer resin layer also holds the three separately fabricated components together and replace any use of extra adhesive.

**Figure 4:**
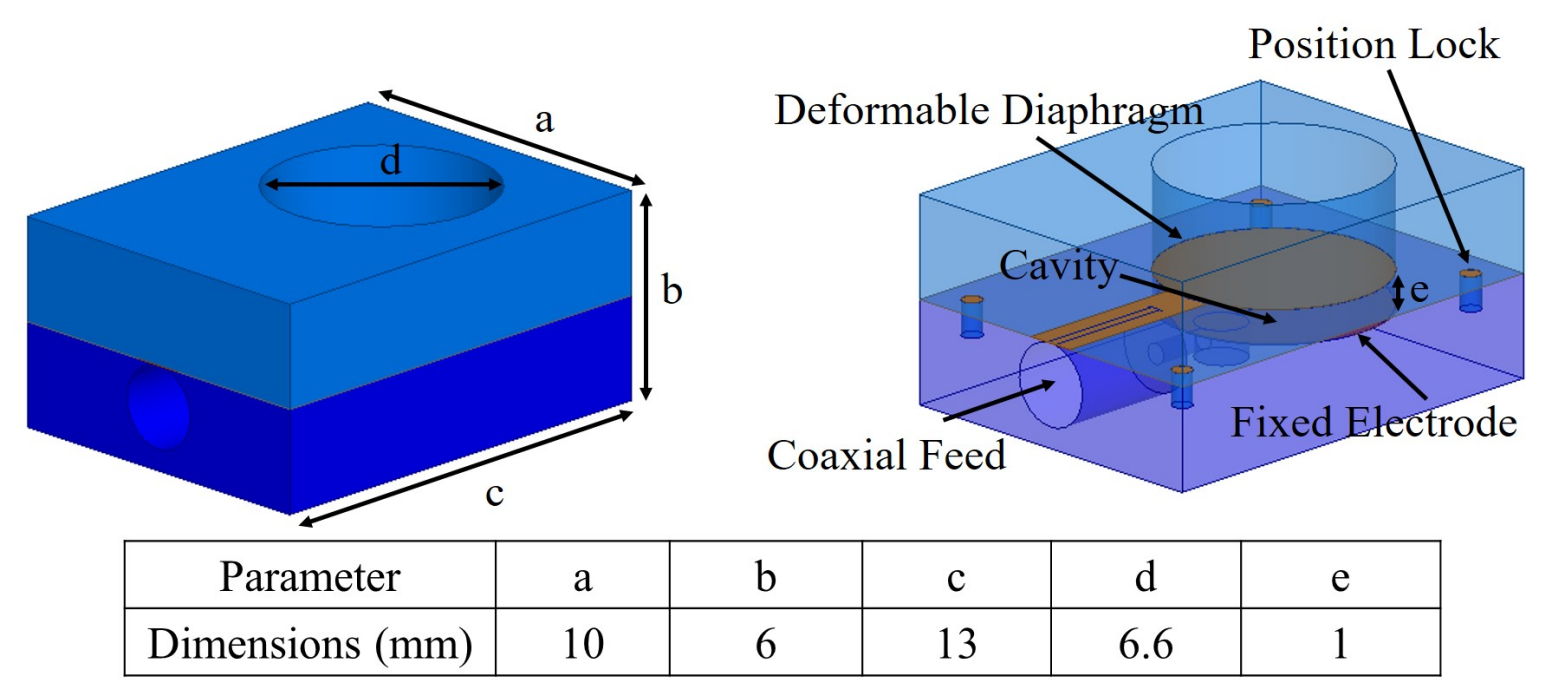
Fully assembled 3D printed pressure sensor with component annotation and dimensions.

Figure 4 shows a fully assembled view of the fabricated pressure sensor, where three components fit perfectly and allow a coaxial cable to make electrical connection with the electrodes for reactance measurement. The total volume of the fabricated sensor is 10 × 13 × 6 mm${}^{3}$. The diameter of the pressure tunnel is 6.6 mm and the depth of the pressure cavity is 1 mm. The change in pressure deforms the LCP electrode and reduces the separation of two electrodes, therefore a change in electrical response, which can be measured using an impedance analyzing equipment.

**Figure 5:**
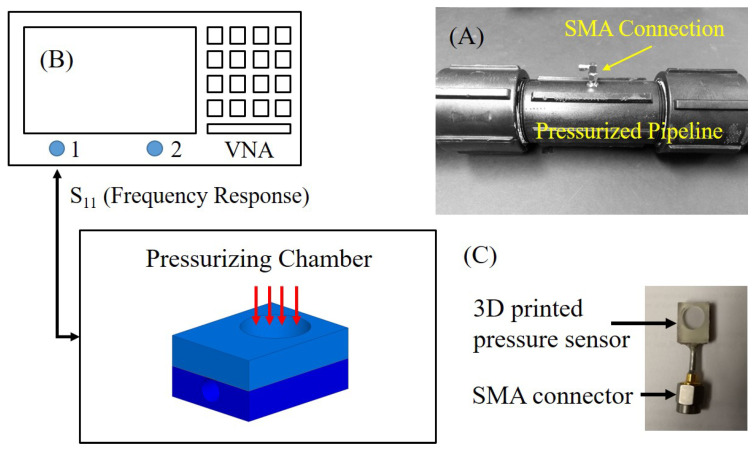
(**A**) Pressurizing chamber (pipe) with integrated pressure sensor, (**B**) Schematic of the measurement setup, and (**C**) Fabricated pressure sensor.

A sealed cavity-based sensor with deformable diaphragm in parallel plate configuration is used to measure the pressure, ranging from 0 to 20 psi. The pressure sensor is integrated inside a polypropylene plastic pipe and probed using an SMA connector from the outside, as shown in Figure 5A. A vector network analyzer (VNA) is used in a direct wired configuration to measure the impedance of the pressure sensor at 2 GHz, as shown in Figure 5B.

**Figure 6:**
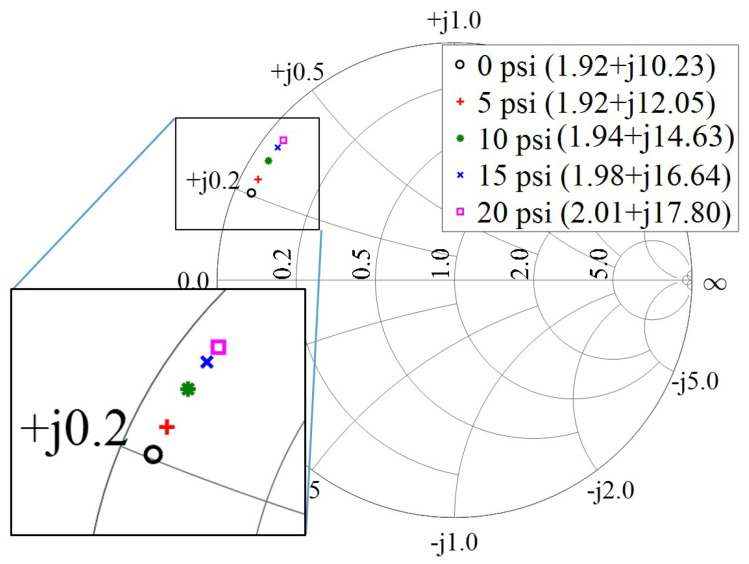
Measured impedance at 2 GHz for pressure range from 0 to 20 psi with a direct wired connection using vector network analyzer (VNA).

The measured impedance is plotted on a normalized smith chart for varying pressure, as shown in Figure 6. The reactive impedance changes from 10.23–17.80 Ω for the applied pressure. A small series resistance is observed in the acquired data, which is due to the parasitics associated with the high frequency and losses from the cable. The direct wired measurements support the hypothesis of significant change in reactive impedance due to pressure difference in between sealed cavity and pipeline pressure. Moreover, it provides an insight into the parasitic real impedance of the sensor and provide data for simulating ideal phase shift for a given range of impedance.

### 2.2. Passive Harmonic RF Sensor Tag

The passive harmonic RF tag uses a reactance-based phase shifting mechanism for wirelessly measuring the change in the applied pressure. The hybrid coupler is used as a phase shifter, which is a four port reciprocal device with an input, output, isolation, and coupled ports. The phase shifter is designed and simulated using ANSYS HFSS^®^ (2019, ANSYS, Inc., Canonsburg, PA, USA) on an FR-4 substrate with dielectric constant ($\epsilon_{r}$) of 4.4, thickness of 1.52 mm and a loss tangent (tan δ) of 0.02. The phase shifter operates at 2 GHz with a 3 dB coupling factor and a 20 dB isolation.

**Figure 7:**
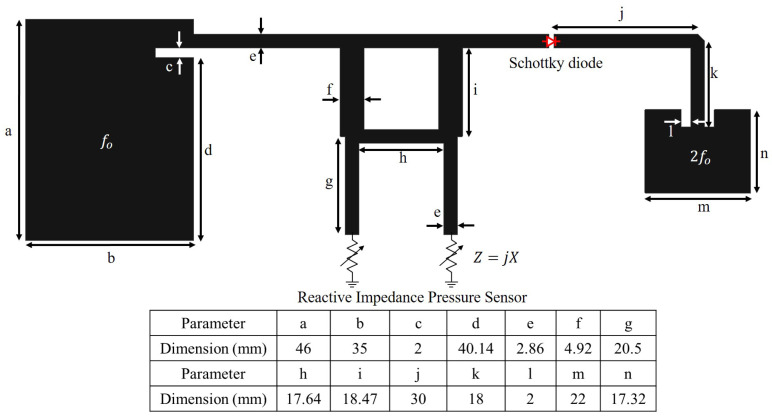
Schematic of the pressure sensing harmonic RF tag along with its dimensions. Signal received using 2 GHz antenna forwarded to the hybrid coupler for phase shift according to the integrated pressure sensor and the second harmonic signal with double shifted phase, generated using diode frequency doubler, is transmitted out using 4 GHz patch antenna.

The phase shifter’s input is connected to a 2 GHz patch antenna for receiving the interrogation signal. The received RF signal is fed to the output and the coupled ports of the coupler, to which a reactive impedance pressure sensor is connected. The RF signal at the output and the coupled ports experience a total internal reflection due to the termination by a reactive sensor element. The internally reflected signal is received from the isolated port without any internal power loss, but with a shifted phase according to the reactance. The isolated port is connected to a non-linear device (Schottky diode), which generate a second harmonic signal at 4 GHz. The Schottky diode (BAT 15-03) doubles the shifted phase along with a frequency. The phase modulated 4 GHz output of the frequency doubler is transmitted back using a patch antenna. The schematic of the designed harmonic RF tag is shown in Figure 7 along with the dimensions.

**Figure 8:**
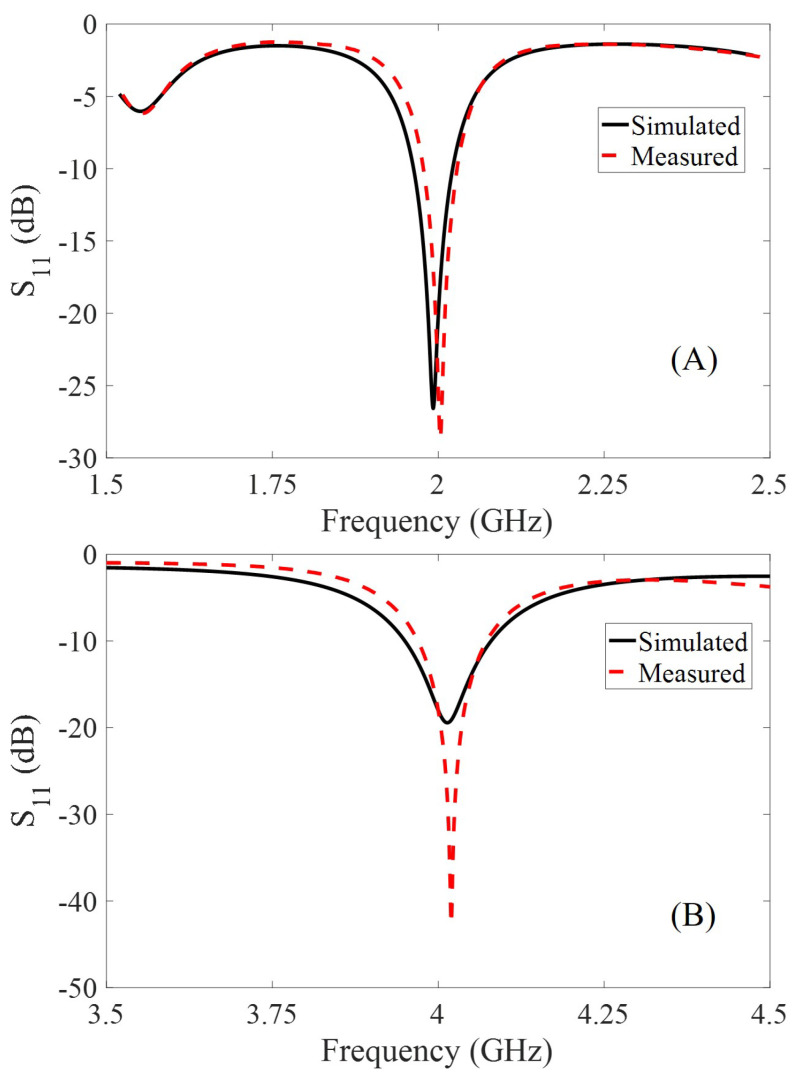
Simulated and measured frequency response; (**A**) Fundamental (2 GHz) and (**B**) Harmonic antenna (4 GHz).

The fundamental and the harmonic antennae are designed in cross-polarization for increasing the SNR and minimizing the interference at the interrogator. The simulated and the measured frequency responses for both the fundamental and the harmonic patch antenna are shown in Figure 8. The measured response closely matches the simulated response, and the observed minimal differences are due to fabrication tolerances.

### 2.3. Harmonic RF Interrogator

**Figure 9:**
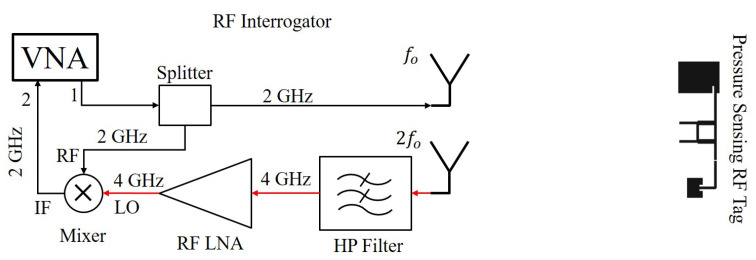
Schematic of the harmonic RF Interrogator consist of a VNA for RF source and phase measurement unit, transmitting and receiving antennas, and miscellaneous RF peripheral circuits for communicating with the harmonic RF sensor tag.

The harmonic RF interrogator is designed to communicate with the passive phase shifting-based RF tag. The interrogator generates an RF signal using a VNA with a signal strength of 13 dBm at 2 GHz and it is transmitted towards the harmonic RF tag using a Vivaldi antenna. The harmonic RF tag receives the fundamental frequency and backscatters the pressure sensor information modulated onto the 4 GHz harmonic signal. The harmonic interrogator receives the modulated signal using a Vivaldi antenna in a cross-polarized configuration. The received harmonic signal is amplified using LNA’s (ZX60-53LNB-S+ and ZX60-43-S+) with a total amplification of 34 dB. The amplified 4 GHz signal is fed into the RF port of the mixer for down converting it to a 2 GHz signal. A reference signal is extracted from the 2 GHz transmission signal using an RF splitter and fed into the LO port of the mixer. The down converted output signal at 2 GHz with the modulated phase information is acquired using VNA at the IF port of the mixer. The schematic of the harmonic RF interrogator communicating with the harmonic RF sensor tag is shown in Figure 9.

## 3. Results

**Figure 10:**
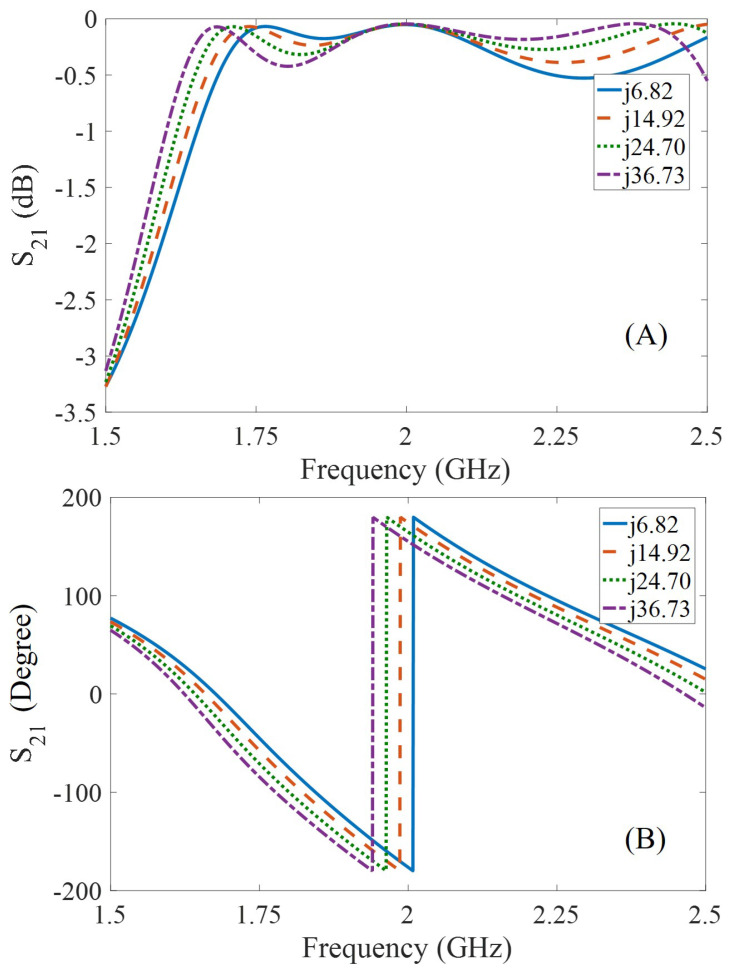
Simulated frequency response of the designed phase shifting hybrid coupler with a variable reactance from 6.82 Ω to 36.73 Ω: (**A**) No power loss is observed at 2 GHz due to changing reactance, (**B**) A total phase shift of 28.55° is observed at 2 GHz due to changing reactance.

Initially, a simulation-based validation study is performed in order to test the phase shifting mechanism using a reactive element. A four port phase shifter is designed in Keysight’s ADS RF simulation software (2017, Keysight Technologies, Santa Rosa, CA, USA ) with a coupled reactive impedance element. The range of reactive impedance is referenced from the previous direct wired measurements shown in Figure 6. The reactive elements are connected to the two of the four ports of hybrid coupler, while using two leftover ports as input and output of 2 GHz signal. The S-parameter study is used in ADS for validating the phase shifting mechanism. The simulated transmission coefficient (S_21_) of the phase shifter is shown in Figure 10. The phase of the transmitted signal at 2 GHz is changed from −176.21° to 155.2° (ΔΦ = 28.55°) with a change in coupled reactance from 6.82–36.73 Ω. The simulated phase shift due to reactive impedance is significant for practical implementation and can be easily detected using a standard phase measurement device. Moreover, the transmitted power at 2 GHz remains unchanged, shows no loss at the operating frequency for the entire range of the changing reactive impedance. Pressure information is modulated on the 2 GHz signal in the form of a phase with no power loss is a significant feat for passive RF tag technology, where a small amount of energy saving can lead to a huge improvement in range or signal-to-noise ratio. In this paper, the no power loss advantage is exploited for better harmonic generation using the Schottky diode, where the frequency conversion losses are inversely proportional to the input power of the signal [24].

**Figure 11:**
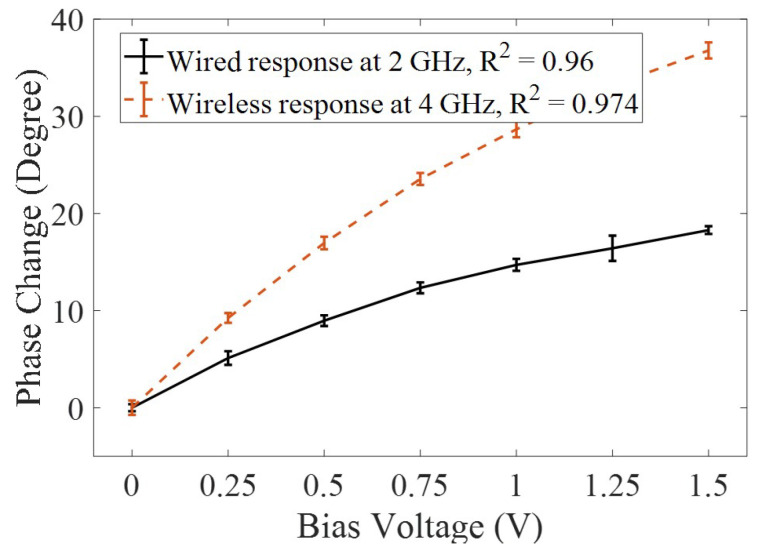
Measured phase change due to variable reactance (introduced with direct current (DC) biasing the varactor diode) in a wired configuration (ΔΦ = 18.28°) using a single frequency approach and wireless configuration (Δ = 36.78°) using the harmonic frequency approach.

The proposed technique is validated by performing two experiments. First, the phase shifter is designed in Ansys HFSS for 2 GHz and fabricated on a FR-4 board for validating the simulation results. The fabricated phase shifter’s response, without a transmitter or a receiver antenna, is measured using the VNA at 2 GHz. A power combiner is used to connect a single reactive element (varactor diode) with the two ports of the phase shifter. The reactive impedance of the varactor diode is varied by applying a bias voltage from 0 V to 1.5 V. The change in phase, due to the change in reactance, of the transmission coefficient (S${}_{21}$) at 2 GHz in direct wired configuration, is shown in Figure 11. The 1.5 V change in bias potential leads to a change of 18.28° in phase. The fabricated phase shifter performed in a similar way to the simulated results. Next, receiver and transmitter patch antennae are connected to the phase shifter with a harmonic doubler and the wireless phase measurement experiment is performed. The harmonic RF sensor tag is placed at a separation of 8 inches from the interrogator antenna for wireless communication. The wirelessly acquired phase change due to applied bias voltage is shown in Figure 11. A phase shift of 36.78° is observed for the wireless configuration, which is approximately double the single frequency wired configuration. In Figure 11, both responses are the average plots of three repetitions and error bars represent the standard deviation between all readings. A constant phase offset with reference to 0° is added in both the wired and the wirelessly acquired data.

Second, a pressurized pipeline setup is designed to test the harmonic RF tag with integrated pressure sensor. A section of polypropylene pipe, 24 inches in length and 2 inches in diameter, is sealed using threaded polypropylene caps.

**Figure 12:**
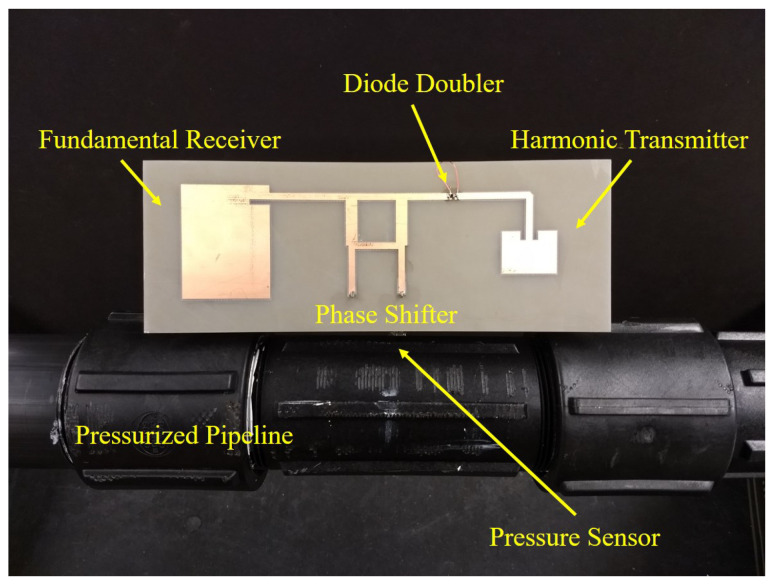
Pressurized polypropylene pipeline setup with the fabricated harmonic RF sensor tag for real-time wireless communication.

An 1/8 inch precision flow valve is connected to both the ends of the pipe for controlling the pressure. A high pressure nitrogen cylinder is used to pressurize the pipe through a compressed yor-lok fitting coupled to a precision flow valve. Due to the safety of research lab and personnel, natural gas pressure pipe application is demonstrated using nitrogen gas. A 20 psi safety valve is connected that sets the limit on the maximum applied pressure. The designed pressure sensor can also work at higher pressure ranges, but due to safety, the upper limit is set to 20 psi for this work. The working principle of the designed sensor is demonstrated for a limited pressure range from 0 to 20 psi. The integrated pressure sensor inside the pipeline is connected to the designed harmonic RF tag, as shown in Figure 12. The harmonic RF tag is capable of receiving RF signal at 2 GHz and modulate the phase of output signal according to the applied pressure.

### Discussion

**Figure 13:**
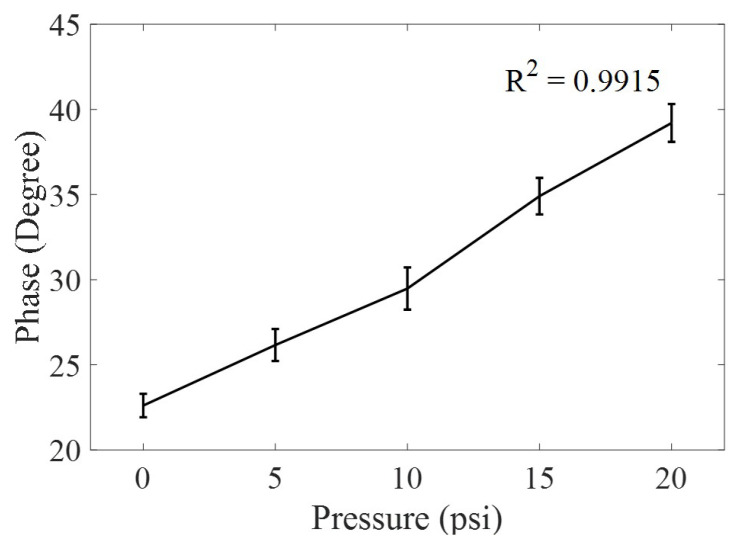
Phase change is wirelessly measured using harmonic RF interrogator due to applied pressure ranging from 0 to 20 psi. The linearity of change in phase due to pressure is represented using coefficient of determination (*R${}^{2}$* = 0.9915).

In our experiment, the applied pressure range is from 0 to 20 psi. Due to applied pressure, the harmonic RF interrogator, placed at 18 inches’ distance from RF tag, measured a phase shift of 16.6° in the received signal at 4 GHz. The wirelessly measured phase is shown in Figure 13, where the measurements are acquired at an interval of 5 psi. All measurements are performed thrice for checking the repeatability and standard deviation at each acquisition point, as shown in Figure 13. The plotted average data of three repetitions show a linearity of 0.9915 (*R${}^{2}$*), which is comparable to existing commercial pressure sensors [25].

The designed harmonic RF sensor tag has a sensitivity of detecting a minimum change of 1 Ω in reactive impedance at 2 GHz and is compatible with other types of reactive impedance based sensors for measuring higher pressure or other physical parameters such as temperature, moisture, etc. The interrogation range of the system can be further enhanced by radiating the maximum allowable power of 4 W, whereas in this work, only 19 mW was used. A maximum communication range of 15 ft can be achieved with 36 dBm (4W) transmitted power at 2 GHz, 20 dB doubler diode conversion loss and −90 dBm receiver sensitivity at 4 GHz.

The 3D printed pressure sensor eliminated the complex fabrication requirement in clean room, while providing similar capability such as piezo-based resistive sensors, MEMS-based capacitive sensor, or FBG-based pressure sensors [7,8,9,10]. The passive wireless feature successfully communicated the pressure information from pipe to interrogator, while eliminating the battery requirement and lowering down the maintenance cost [18].

## 4. Conclusions

In this paper, a low cost 3D pressure sensor and passive wireless sensing mechanism is presented for monitoring the structural integrity of the gas pipelines. The developed harmonic pressure sensing system has a good sensitivity (1 Ω) towards reactive impedance, better linearity (R${}^{2}$ = 0.9915), a higher SNR, lower modulation losses, and a longer communication range. The harmonic communication eliminates the self-jamming, reduces the multi-path interference, and doubles the measurement sensitivity (phase change) due to applied pressure. The coupler-based phase shifting mechanism efficiently modulated the pressure information on carrier harmonic frequency. The demonstrated detection range of applied pressure from 0 to 20 psi is practical for natural gas distribution and main pipeline infrastructure.

Furthermore, the logical next step of this research is to expand the application to higher pressure ranges and study the performance of sensor in liquid medium while focusing on the effects of non-dissolved particles. The developed long range wireless sensing platform allows a continuous, safe, reliable, and real-time monitoring of the pipeline and provides a cost-efficient solution for operations in refineries and chemical plants.
